# Frequency and Influencing Factors of Rubber Dam Usage in Tianjin: A Questionnaire Survey

**DOI:** 10.1155/2016/7383212

**Published:** 2016-07-31

**Authors:** Huiru Zou, Yanni Li, Xiaoli Lian, Yan Yan, Xiaohua Dai, Guanhua Wang

**Affiliations:** Research Center of Tianjin Stomatological Hospital, 75 Dagu Road, Heping District, Tianjin 300041, China

## Abstract

*Objective*. To investigate the frequency and influencing factors of rubber dam usage for endodontic procedures among general dentistry practitioners and specialized practitioners (endodontist) in Tianjin.* Methods*. Three hundred questionnaires were distributed among practitioners from 3 different types of medical institutions in Tianjin. Data were collected and analysed using Chi-square tests.* Results*. There were 63.3% of respondents who have used rubber dam (response rate 82.7%, valid response rate 76.3%). However, only 0.4% and 3.1% of them recognized using rubber dam “every time” during caries direct restoration and root canal therapy, respectively. There was no significant difference in rubber dam usage between male and female practitioners. Among the respondents, practitioners with working experience between 5 and 10 years showed the highest usage rate (76.3%), while practitioners working more than 20 years showed the lowest (53.2%). The endodontists gained the highest and the most frequent usage rate and the best rubber dam technique mastering skills. Practitioners working in those stomatological departments of general hospitals showed the lowest rubber dam usage rate.* Conclusions*. The prevalence of rubber dam usage in Tianjin city is still low. The practitioner's gender, years of professional experience, general or specialized field, and the type of dental setting they work for are the factors that need to be considered during making policy and executing training.

## 1. Introduction

Since being introduced by Dr. Barnum in the 1860s, rubber dam isolation technique has gradually gained recognition as an essential technique in the process of dental treatments. The benefits of rubber dam are well known, which can effectively protect patients and doctors and provide a more professional, safe, and comfortable medical experience. However, various prevalences of rubber dam usage have been reported from different countries [1–6]. In China, little data on utilization rate is available. This study conducted a questionnaire survey of 300 dentists from 18 medical institutional dental settings in Tianjin, in order to determine the overall attitude of dentists towards rubber dam application, explore possible influencing factors such as the practitioner's gender, years of professional experience, general or specialized field, and the type of medical institutional dental settings they work for, and hopefully make some contribution in policies development on how to promote the rubber dam technique usage in dental practice in China.

## 2. Materials and Methods

### 2.1. Dentists Recruited

Three hundred general dentists and endodontists from 2 stomatological hospitals, 11 stomatological departments of general hospitals, and 5 private hospitals were selected in this questionnaire survey. Inclusive criteria were as follows: all employed practitioners from above institutions, including graduate intern. Undergraduate and college students were excluded from this study.

### 2.2. Survey Content

The questionnaire consisted of 30 questions concerning different aspects of rubber dam usage, such as (1) the basic information regarding the practitioner's gender, years of professional experience, general or specialized field, and the type of medical institutional settings they work for, (2) rubber dam awareness, source of knowledge gained for rubber dam usage, and prevalence in caries filling and root canal treatment application, and (3) the time required for the clinical installation of rubber dam and problems occurring during rubber dam usage. This survey divided the mastery degree of rubber dam technique into 4 levels as 0, 1, 2, and 3. Level 0 means totally having no knowledge of rubber dam technique. Level 1 means having some knowledge of rubber dam technique, but having no practical experience. Level 2 means knowing some and handling easy situations but not dealing with complex cases. Level 3 means fully mastering rubber dam technique and handling any situations without problem. Practitioners were asked to make self-assessment of the extent of their rubber dam technique skills. This study mainly analysed the possible effects of influencing factors such as the practitioner's gender, years of professional experience, field and the type of medical institutions on the mastery degree, and prevalence of rubber dam usage.

### 2.3. Survey Method and Statistical Analysis

All questions had 2 or more different choices. Respondents were informed to choose one or more suitable answers. Three persons were entrusted for handling out and collecting the questionnaires. Questionnaire with unanswered questions were treated as invalid questionnaire. All data collected from the questionnaire survey were entered into Microsoft Excel using a double entry method. SPSS17.0 statistical software was used for statistical analysis. Frequencies and percentage distributions were analysed using Chi-square test to investigate the influence of gender, years of professional experience, and field and type of medical institutions on rubber dam usage. The chosen level of statistical significance was set at *P* < 0.05.

## 3. Results

**Figure 1:**
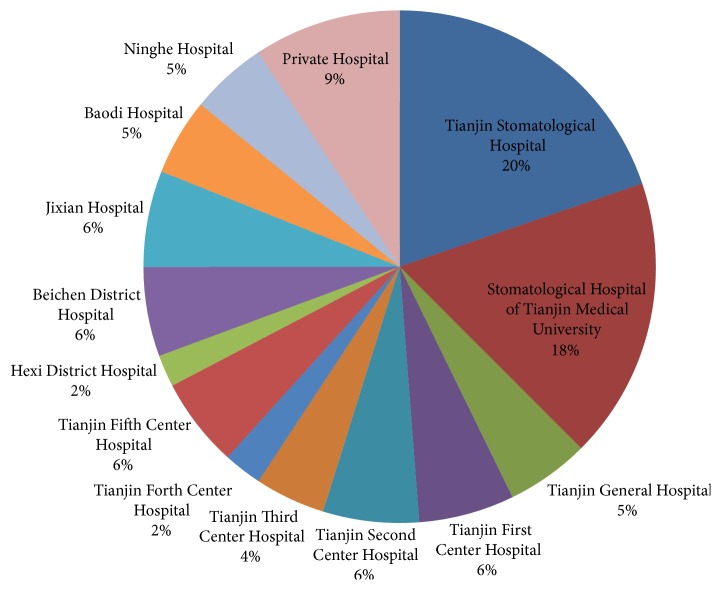
The distribution of medical institutions and the recovery of respondents.

**Figure 2:**
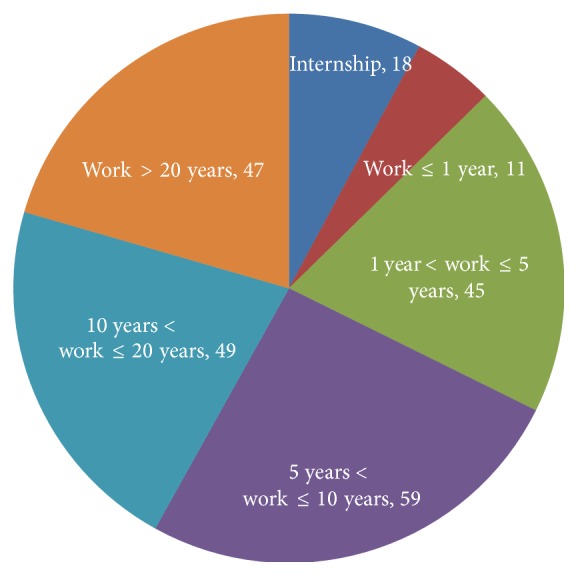
The distribution of respondents' years of working experience.

**Figure 3:**
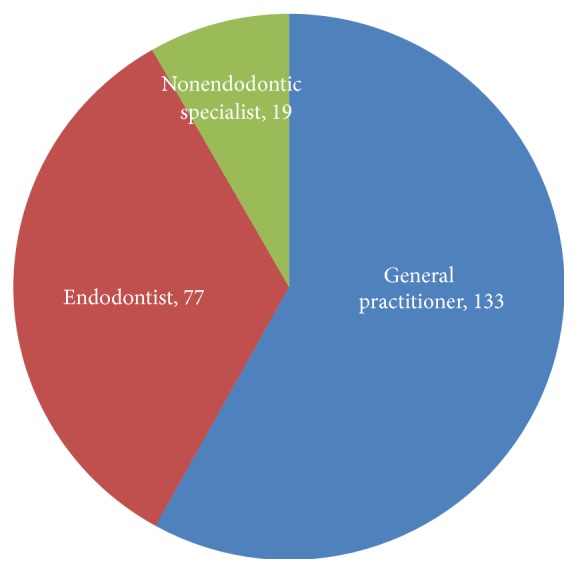
The field (general or specialized) distribution of respondents engaged in dentistry.

**Figure 4:**
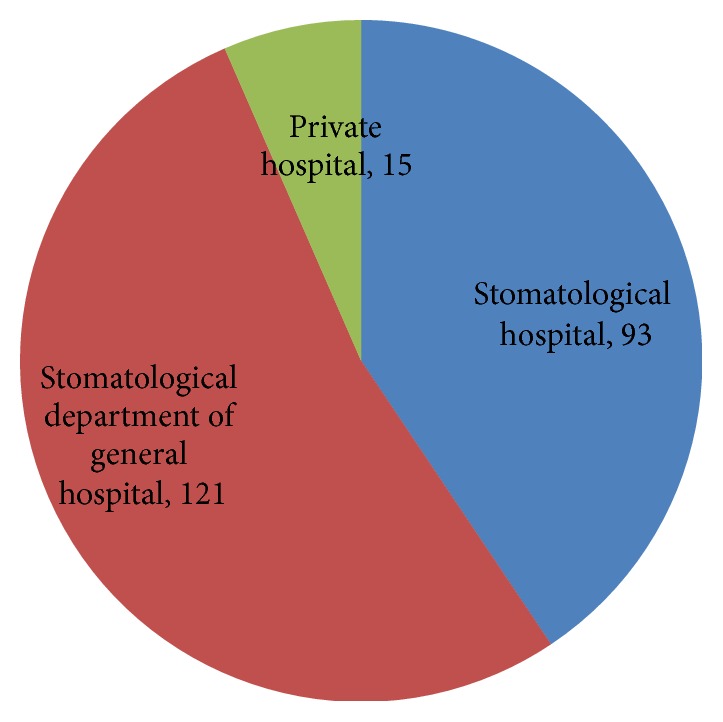
The distribution of the type of medical institutions where respondents work.

Eighteen medical institutional dental settings in Tianjin City were selected in this questionnaire survey study. Three hundred surveys were distributed; a total of 248 questionnaires were collected back (the questionnaire recovery rate was 82.7%); and 229 of the 248 returned surveys are valid questionnaires (the valid response rate is 76.3%). The distribution of the medical institution and the recovery of the respondents in the survey are shown in Figure 1. Altogether, 102 (44.5%) were males, while 127 (55.5%) were females. The working experience of respondents was shown in Figure 2. The field (general or specialized) of practice scope of respondents who engaged in dentistry was shown in Figure 3. The distribution of the type of medical institutions where respondents work was shown in Figure 4.

There were 63.3% of respondents having used rubber dam. However, only 0.4% and 3.1% of practitioners (general dentists versus endodontists) recognized that they always use rubber dam during dental caries direct restoration and root canal therapy, respectively; 45.4% and 39.7% of them “never” use rubber dam during these procedures, respectively. 14% of respondents were of “Level 0,” 40.6% of “Level 1,” and 34.9% of “Level 2” in their self-assessment of rubber dam technique mastery degree; only 10.5% of respondents marked themselves as Level 3.

Table 1 showed that 68.6% of male respondents and 59.1% of female respondents have used rubber dam. There was no significant difference between different genders (*P* > 0.05). Refined to the caries filling treatment and root canal treatment, there was also no significant difference of rubber dam usage (*P* > 0.05).

76.3% of 5 years < work ≤ 10 years respondents have used rubber dam which was the highest rate, while work > 20 years respondents showed the lowest (53.2%). But there were no significant differences (*P* > 0.05) (Table 2).

Tables 3 and 4 showed that there were significant differences among general or specialized fields and the type of medical institutions in rubber dam usage rates (*P* < 0.01). The endodontic specialists gained the highest and the most frequently usage rate and the best rubber dam technique mastering skills. Respondents' self-assessment results also showed significant differences among different medical institutions (*P* < 0.01).

## 4. Discussion

Rubber dam is considered an ideal device for tooth isolation. The use of rubber dam provides a significantly higher success rate during restorative procedures and root canal treatment [7]. In recent years, the application of rubber dam isolation technique in dental treatment is becoming more and more widely spread. However, the level and development of its usage around the world are still uneven. A survey investigating general practitioners in the United States showed that 60% respondents always use rubber dam, 16% usually use it, 13% sometimes use it, and 11% never use it [6]. After surveying 1716 eligible general dentists, Lawson et al. reported that 697 (47%) always use rubber dam, while this percentage varied by tooth type [8]. A survey investigated general practitioners, specialized practitioners, undergraduate final year students, and endodontists in the state of Odisha, India. The results showed that 30% have used rubber dam for root canal cases and 23% use them for all cases of root canal treatment. Rubber dam usage was 15.4% in paediatric patients and 34.4% in adult patients [5]. The prevalence of rubber dam use was only 18% in southern Nigeria. Up to 77% of the respondents had not used rubber dams or did not know how to use them [9]. Rubber dam usage rate is less than 8% in the Republic of Czech [10]. In China, the situation of rubber dam usage is also far from being good and optimistic. There are few surveys investigating rubber dam usage rate in Chinese mainland. Therefore, this study investigated 300 general practitioners and specialists from 18 medical institutions of Tianjin city. The results showed that 63.3% of respondents used rubber dam; however, only 0.4% and 3.1% always use rubber dam in the treatment of cavity filling and root canal treatment, respectively. The overall usage of rubber dam technique is really low. Compared with the developed countries, there is still a big gap.

Several factors may influence the usage of rubber dam, such as the practitioner's gender, years of professional experience, field, type of medical institutions, costs and profits, and previous experience in using rubber dam. Unal et al. reported that gender affected the preference of intracanal medicament, periapical radiographs for working-length determination, root canal instrument, root canal sealers, and root canal obturation technique. However, there was no statistical significance (*P* > 0.05) showing that gender affects rubber dam usage [11]. The survey carried out in India also found that there was no significant difference between males and females on rubber dam usage [12]. Our results compare well with these studies mentioned above.

Years of working experience is considered to be closely related to the usage of rubber dam technique. Savani et al. reported that general practitioners (GPs) within 10 years of experience were more likely to use rubber dam compared with those in practice for more than 20 years (*P* < 0.05). Interestingly, GPs with more than 5 hours of continuing education (CE) were more likely to use rotary instrumentation, irrigant activation devices, and apex locators and perform molar root canal therapy and retreatment but not likely to use rubber dam [6]. Gupta and Rai showed that respondents with less than 5 years of working experience use rubber dam more than those with more than 15 years of working experience. There were significant differences between these two groups [12]. This study shows that dentists who have 5 years < working experience ≤ 10 years gain the highest rubber dam usage rate while those working more than 20 years have the lowest usage rate. Savani et al. pointed out that the more recently graduated dentists were more likely to use rubber dam. They were more likely to adopt new technologies than those who practiced for more than 20 years [6]. Anabtawi et al. showed that even the graduation year was not statistically significant in multivariable regressions when including the “type of practice” variable, and higher usage rate among newly graduates had been confirmed [1].

However, there are also opposite reports that older practitioners use rubber dam more often than their younger counter parts [13]. While in that study, the majority of practitioners did not use rubber dam during root canal therapy. Another report showed that years of working experience did not influence the preference of isolation methods [11]. Practitioners are more likely to do what they have been taught in dental school after their graduation. When they have never used rubber dam in their studying life, they tend to not try to use it later. Maybe that is the reason why studies showed that more experienced clinicians were resistant to use rubber dam. Furthermore, postgraduation training is also important and may influence rubber dam usage. A significantly higher proportion of respondents having postgraduate qualifications carried out root canal treatment using rubber dam, compared to those who did not have postgraduate qualifications [12]. Dentists with postgraduation training placed rubber dam more frequently [14], while endodontic practice of general dentists sometimes did not always comply with quality guidelines [4]. Several studies showed that rubber dam is used frequently for root canal treatment compared to operative treatment. In this study, data also showed low prevalence of its usage during endodontic therapy. Therefore, further emphasis should be placed on the advantages of rubber dam usage in clinical dentistry at dental school and through continuing dental education for practitioners to update their knowledge [5].

Many studies have shown that level of specialization affected dentists' rubber dam usage. Endodontists used more frequently rubber dam in restorative dentistry or root canal treatment than did nonspecialists [1, 15]. This study showed significant differences among general practitioners, endodontists, and nonendodontic specialists in the prevalence of rubber dam usage. Rubber dam usage rate among endodontists was much higher than that of general dentists and other nonendodontists, similar to other reports in the literature.

The type of medical institutions also has certain impacts on rubber dam usage. Rubber dam usage varied significantly by geographic region and practice type [1]. Udoye and Jafarzadeh reported that dentists in the government sector used rubber dam more often than did dentists in the private sector [9]. Lin et al. found that rubber dam usage in public hospital was significantly higher than that of private dental clinics in Taiwan [16]. Practitioners in group practices used rubber dam more than those in solo practices [17]. This survey shows that the use of rubber dam in the department of stomatology of general hospital and private clinic is relatively low compared with specialized stomatological hospital. Interestingly, as shown in Table 4, although there was lower percentage (47.9%) in general hospital group for “have used rubber dam” compared with that (80.5%) in specialized stomatological hospital group, the percentage of “often use” plus “always use” in general hospital group in root canal treatment was much higher (46.8% + 6.5%) compared with that (13.5% + 1.5%) in specialized hospital group, while specialized hospital group owned higher rubber dam usage rate in caries filling compared with other groups. It is suggested that special-tailored training should be strengthened for dentist working at different types of medical institutions.

Other factors such as ethnicity, dental insurance, and age and patient level characteristics may also influence the prevalence of rubber dam usage [18]. Therefore, although most of dentists agree that rubber dam is the standard of care in endodontic treatments, discrepancies still existed between principles and practice. Emphasis on education and increased awareness of the importance of rubber dam usage are needed. Dentists should update their knowledge and practices with current techniques and materials through continuous dental education programs [19]. The current guidelines need to be more clear and straightforward and special management should be highlighted [20].

## 5. Conclusion

The survey indicated that the prevalence of rubber dam usage in Tianjin city is still low. The practitioner's gender, years of professional working experience, general or specialized field, and the type of medical institutional settings they work for are factors that need to be considered during making policy and executing training.

## Figures and Tables

**Table 1:** Respondents' gender and rubber dam usage.

	Total	Male	Female
Have used rubber dam			
Yes	145 (63.3%)	70 (68.6%)	75 (59.1%)
No	84 (36.7%)	32 (31.4%)	52 (40.9%)

Use rubber dam in caries filling			
Never use	104 (45.4%)	43 (42.2%)	61 (48.0%)
Occasionally use	87 (38.0%)	41 (40.2%)	46 (36.2%)
Often use	37 (16.2%)	17 (16.7%)	20 (15.7%)
Always use	1 (0.4%)	1 (1.0%)	0 (0.0%)

Use rubber dam in root canal treatment			
Never use	91 (39.7%)	37 (36.3%)	54 (42.5%)
Occasionally use	77 (33.6%)	40 (39.2%)	37 (29.1%)
Often use	58 (25.3%)	22 (21.6%)	36 (28.3%)
Always use	7 (3.1%)	3 (2.9%)	4 (3.1%)

Mastery degree of rubber dam usage			
Level 0	32 (14.0%)	12 (11.8%)	20 (15.7%)
Level 1	93 (40.6%)	46 (45.1%)	47 (37.0%)
Level 2	80 (34.9%)	33 (32.4%)	47 (37.0%)
Level 3	24 (10.5%)	11 (10.8%)	13 (10.2%)

Total	229	102	127

**Table 2:** Respondents' years of working experience and rubber dam usage.

	Total	Internship	Work ≤ 1 year	1 year < work ≤ 5 years	5 years < work ≤ 10 years	10 years < work ≤ 20 years	Work > 20 years
Have used rubber dam							
Yes	145 (63.3%)	10 (55.6%)	8 (72.7%)	26 (57.8%)	45 (76.3%)	31 (63.3%)	25 (53.2%)
No	84 (36.7%)	8 (44.4%)	3 (27.3%)	19 (42.2%)	14 (23.7%)	18 (36.7%)	22 (46.8%)

Use rubber dam in caries filling							
Never use	104 (45.4%)	10 (55.6%)	3 (27.3%)	22 (48.9%)	19 (32.2%)	24 (49.0%)	26 (55.3%)
Occasionally use	86 (37.6%)	8 (44.4%)	7 (63.6%)	19 (42.9%)	25 (42.4%)	15 (30.6%)	12 (25.5%)
Often use	38 (16.6%)	0 (0.0%)	1 (9.1%)	4 (8.9%)	15 (25.4%)	10 (20.1%)	8 (17.0%)
Always use	1 (0.4%)	0 (0.0%)	0 (0.0%)	0 (0.0%)	0 (0.0%)	0 (0.0%)	1 (2.1%)

Use rubber dam in root canal treatment							
Never use	91 (39.7%)	9 (50.0%)	4 (36.4%)	18 (40.0%)	14 (23.7%)	23 (46.9%)	23 (48.9%)
Occasionally use	77 (33.6%)	8 (44.4%)	5 (45.5%)	17 (37.8%)	20 (33.9%)	13 (26.5%)	14 (29.8%)
Often use	54 (23.6%)	1 (5.6%)	2 (18.2%)	10 (22.2%)	22 (37.3%)	11 (22.4%)	8 (17.0%)
Always use	7 (3.1%)	0 (0.0%)	0 (0.0%)	0 (0.0%)	3 (5.1%)	2 (4.1%)	2 (4.3%)

Mastery degree of rubber dam usage							
Level 0	32 (14.0%)	2 (11.1%)	1 (9.1%)	6 (13.3%)	7 (11.9%)	5 (10.2%)	11 (23.4%)
Level 1	93 (40.6%)	13 (72.2%)	8 (72.7%)	18 (40.0%)	14 (23.7%)	22 (444.9%)	18 (38.3%)
Level 2	80 (34.9%)	2 (11.1%)	2 (18.2%)	17 (37.8%)	29 (49.2%)	16 (32.7%)	14 (29.8%)
Level 3	24 (10.5%)	1 (5.6%)	0 (0.0%)	4 (8.9%)	9 (15.3%)	6 (12.2%)	4 (8.5%)

Total	229	18	11	45	59	49	47

**Table 3:** Respondents' clinical specialty and rubber dam usage.

	Total	General	Specialized	
			Endodontic	Nonendodontic
Have used rubber dam				
Yes	145 (63.3%)	75 (56.4%)	63 (81.8%)	7 (36.8%)
No	84 (36.7%)	58 (43.6%)	14 (18.2%)	12 (63.2%)

Use rubber dam in caries filling				
Never use	104 (45.4%)	75 (54.1%)	17 (22.1%)	15 (78.9%)
Occasionally use	86 (37.6%)	49 (36.8%)	33 (42.9%)	4 (21.1%)
Often use	38 (16.6%)	11 (8.3%)	27 (35.1%)	0 (0.0%)
Always use	1 (0.4%)	1 (0.8%)	0 (0.0%)	0 (0.0%)

Use rubber dam in root canal treatment				
Never use	91 (39.7%)	64 (48.1%)	13 (16.9%)	14 (73.7%)
Occasionally use	77 (33.6%)	49 (36.8%)	23 (29.9%)	5 (26.3%)
Often use	54 (23.6%)	18 (13.5%)	36 (46.8%)	0 (0.0%)
Always use	7 (3.1%)	2 (1.5%)	5 (6.5%)	0 (0.0%)

Mastery degree of rubber dam usage				
Level 0	32 (14.0%)	23 (17.3%)	4 (5.2%)	5 (26.3%)
Level 1	93 (40.6%)	70 (52.6%)	13 (16.9%)	10 (52.6%)
Level 2	80 (34.9%)	34 (25.6%)	42 (54.5%)	4 (21.1%)
Level 3	24 (10.5%)	6 (4.5%)	18 (23.4%)	0 (0.0%)

Total	229	133	77	19

**Table 4:** The type of medical institutions where respondents work and rubber dam usage.

	Total	Specialized hospital	General hospital	Private hospital
Have used rubber dam				
Yes	145 (63.3%)	75 (80.6%)	58 (47.9%)	12 (80.0%)
No	84 (36.7%)	18 (19.4%)	63 (52.1%)	3 (20.0%)

Use rubber dam in caries filling				
Never use	104 (45.4%)	21 (22.6%)	80 (66.1%)	3 (20.0%)
Occasionally use	86 (37.6%)	49 (52.7%)	30 (24.8%)	7 (46.7%)
Often use	38 (16.6%)	23 (24.7%)	11 (9.1%)	4 (26.7%)
Always use	1 (0.4%)	0 (0.0%)	0 (0.0%)	1 (6.7%)

Use rubber dam in root canal treatment				
Never use	91 (39.7%)	64 (48.1%)	13 (16.9%)	14 (73.7%)
Occasionally use	77 (33.6%)	49 (36.8%)	23 (29.9%)	5 (26.3%)
Often use	54 (23.6%)	18 (13.5%)	36 (46.8%)	0 (0.0%)
Always use	7 (3.1%)	2 (1.5%)	5 (6.5%)	0 (0.0%)

Mastery degree of rubber dam usage				
Level 0	32 (14.0%)	7 (7.5%)	19 (15.7%)	6 (40.0%)
Level 1	93 (40.6%)	28 (30.1%)	60 (49.6%)	5 (33.3%)
Level 2	80 (34.9%)	37 (39.8%)	39 (32.2%)	4 (26.7%)
Level 3	24 (10.5%)	21 (22.6%)	3 (2.5%)	0 (0.0%)

Total	229	93	121	15
